# Phase Ib study of pevonedistat, a NEDD8-activating enzyme inhibitor, in combination with docetaxel, carboplatin and paclitaxel, or gemcitabine, in patients with advanced solid tumors

**DOI:** 10.1007/s10637-018-0610-0

**Published:** 2018-05-21

**Authors:** A Craig Lockhart, Todd M. Bauer, Charu Aggarwal, Carrie B. Lee, R Donald Harvey, Roger B. Cohen, Farhad Sedarati, Tsz Keung Nip, Hélène Faessel, Ajeeta B. Dash, Bruce J. Dezube, Douglas V. Faller, Afshin Dowlati

**Affiliations:** 1 Division of Medical Oncology, University of Miami/Sylvester Comprehensive Cancer Center, 1120 NW 14th Street, Suite 650L, Miami, FL USA; 2 Drug Development, Sarah Cannon Research Institute/Tennessee Oncology PLLC., Nashville, TN USA; 3 Division of Hematology-Oncology, Department of Medicine, Perelman Center for Advanced Medicine, University of Pennsylvania, Philadelphia, PA USA; 4 Division of Hematology and Oncology, University of North Carolina at Chapel Hill, Chapel Hill, NC USA; 5 Departments of Hematology and Medical Oncology and Pharmacology, Emory University School of Medicine, Winship Cancer Institute of Emory University, Atlanta, GA USA; 6 Department of Medicine, Perelman Center for Advanced Medicine, University of Pennsylvania, Philadelphia, PA USA; 7 Oncology Clinical Research, Millennium Pharmaceuticals, Inc., Cambridge, MA USA; 8 Biostatistics, Takeda Development Centre EU, London, UK; 9 Quantitative Clinical Pharmacology, Millennium Pharmaceuticals, Inc., Cambridge, MA USA; 10 Translational and Biomarker Research, Millennium Pharmaceuticals, Inc., Cambridge, MA USA; 11 Department of Medicine, Case Western Reserve University, Cleveland, OH USA

**Keywords:** Pevonedistat, Standard-of-care chemotherapies, Advanced solid tumors, Clinical research, Phase Ib study

## Abstract

*Purpose* This phase Ib study (NCT01862328) evaluated the maximum tolerated dose (MTD), safety, and efficacy of pevonedistat in combination with standard-of-care chemotherapies in patients with solid tumors. *Methods* Patients received pevonedistat with docetaxel (arm 1, *n* = 22), carboplatin plus paclitaxel (arm 2, *n* = 26), or gemcitabine (arm 3, *n* = 10) in 21-days (arms 1 and 2) or 28-days (arm 3) cycles. A lead-in cohort (arm 2a, *n* = 6) determined the arm 2 carboplatin dose. Dose escalation proceeded via continual modified reassessment. *Results* Pevonedistat MTD was 25 mg/m^2^ (arm 1) or 20 mg/m^2^ (arm 2); arm 3 was discontinued due to poor tolerability. Fifteen (23%) patients experienced dose-limiting toxicities during cycle 1 (grade ≥3 liver enzyme elevations, febrile neutropenia, and thrombocytopenia), managed with dose holds or reductions. Drug-related adverse events (AEs) occurred in 95% of patients. Most common AEs included fatigue (56%) and nausea (50%). One drug-related death occurred in arm 3 (febrile neutropenia). Pevonedistat exposure increased when co-administered with carboplatin plus paclitaxel; no obvious changes were observed when co-administered with docetaxel or gemcitabine. Among 54 response-evaluable patients, two had complete responses (arm 2) and 10 had partial responses (three in arm 1, one in arm 2a, six in arm 2); overall response rates were 16% (arm 1) and 35% (arm 2). High ERCC1 expression correlated with clinical benefit in arm 2. *Conclusion* Pevonedistat with docetaxel or with carboplatin plus paclitaxel was tolerable without cumulative toxicity. Sustained clinical responses were observed in pretreated patients receiving pevonedistat with carboplatin and paclitaxel. ClinicalTrials.gov identifier: NCT01862328.

**Electronic supplementary material:**

The online version of this article (10.1007/s10637-018-0610-0) contains supplementary material, which is available to authorized users.

## Introduction

The ubiquitin-proteasome system regulates turnover of proteins involved in apoptosis, proliferation, and signal transduction [1–3]. Cullin-RING ligases (CRLs), the largest subfamily of E3 ubiquitin ligases, target proteins for proteasomal degradation through the addition of polyubiquitin chains [2]. CRL activation via conjugation to the small ubiquitin-like protein NEDD8 (neural precursor cell expressed, developmentally downregulated 8) is regulated by NEDD8-activating enzyme (NAE). CRLs and associated regulatory proteins are attractive novel targets for the development of antitumor agents [2, 3].

Pevonedistat (TAK-924/MLN4924) is a first-in-class, investigational, small-molecule NAE inhibitor that covalently binds to NEDD8, leading to apoptotic cell death. Pevonedistat has shown single-agent activity in various human solid tumor cell lines and xenograft models, as well as in patients with advanced solid tumors and hematologic malignancies [2, 4–12]. Pevonedistat delays completion of platinum-induced DNA repair in a transcription-coupled nucleotide excision repair (NER) and interstrand crosslink repair (ICR) pathway−dependent mechanism [13]. Excision repair cross-complementation group 1 (ERCC1), a component of the NER pathway, is thought to be involved in resistance to platinum-based therapies [14, 15] and predictive of decreased efficacy of platinum therapy [16–21]. Pevonedistat enhances the antitumor activity of taxanes and gemcitabine, is synergistic with platinum salts, and is active in platinum-resistant tumors [13, 22–24].

The safety and activity of pevonedistat monotherapy were investigated in a phase I study in patients with acute myeloid leukemia (AML) or myelodysplastic syndromes (MDS), which demonstrated that treatment was feasible with clinical activity [11]. Additionally, the efficacy and safety of pevonedistat in combination with the standard-of-care agent azacitidine have been investigated in treatment-naïve patients with AML (ClinicalTrials.gov identifier NCT01814826) [25]. Phase II and III studies of pevonedistat in combination with azacitidine in high-risk MDS, chronic myelomonocytic leukemia, or low-blast count AML are currently ongoing (ClinicalTrials.gov identifiers NCT02610777 and NCT03268954). To further explore the role of pevonedistat in combination with standard-of-care chemotherapies, this phase Ib study aimed to determine the maximum tolerated dose (MTD) and assess the safety, tolerability, and pharmacokinetics of pevonedistat in combination with docetaxel, carboplatin plus paclitaxel, or gemcitabine in patients with advanced solid tumors. The expression of ERCC1 as a candidate biomarker of response to treatment with pevonedistat in combination with carboplatin plus paclitaxel therapy was also investigated.

## Methods

### Study design and patients

This was an open-label, multicenter, phase Ib, dose-escalation study of pevonedistat in combination with either docetaxel (arm 1), carboplatin plus paclitaxel (arm 2), or gemcitabine (arm 3) in adult patients with confirmed solid tumors who had progressed despite standard therapy or for whom conventional therapy was considered ineffective (ClinicalTrials.gov identifier NCT01862328; Online Resource: Supplementary Fig. 1 a). Patient eligibility criteria are described in the Online Resource. Patients were enrolled between 10 June 2013 and 04 May 2015 at six US centers. This study was approved by the Institutional Review Board and Independent Ethics Committee at each site and conducted in accordance with all applicable regulatory requirements, Good Clinical Practice standards, and the Declaration of Helsinki. All patients provided written informed consent.

Dose escalation in arm 1 consisted of intravenous pevonedistat at a 15 mg/m^2^ starting dose on days 1, 3, and 5 plus docetaxel 75 mg/m^2^ on day 1 of 21-day cycles. A lead-in cohort (arm 2a) of six patients received pevonedistat 15 mg/m^2^ on days 1, 3, and 5 plus carboplatin AUC_6_ (target area under the concentration-time curve of 6 mg/mL·min) on day 1 to determine the dose of carboplatin in arm 2. If two or more dose-limiting toxicities (DLTs) were reported, carboplatin dosing was to be reduced to AUC_5_, and paclitaxel be reduced from the planned 200 to 175 mg/m^2^. Patients in arm 2 received escalating doses of pevonedistat starting at 15 mg/m^2^ on days 1, 3, and 5, in combination with carboplatin AUC_5_ plus paclitaxel 175 mg/m^2^ on day 1 of 21-day cycles. In arm 3, patients received escalating doses of pevonedistat starting at 25 mg/m^2^ and gemcitabine 1000 mg/m^2^ on days 1, 8, and 15 of 28-day cycles. Planned pevonedistat doses were higher in arm 3 (versus arm 1 and arm 2) but administration was on a weekly schedule (versus three times a week in arm 1 and arm 2) to match the dosing schedule of gemcitabine, to ensure exposure to both drugs and leverage the mechanism of action of the two drugs. Full dosing details are included in the Online Resource.

Pevonedistat dose escalation (Online Resource: Supplementary Fig. 1 b) proceeded via an adaptive Bayesian continual reassessment method (CRM) based on cycle 1 DLTs (Online Resource). Upon MTD determination in each arm, additional patients were enrolled to confirm the MTD (MTD expansion cohorts). Patients were treated for up to 12 cycles or until disease progression or unacceptable toxicity. Patients with clinical benefit could continue with combination treatment or single-agent pevonedistat beyond 12 cycles.

The primary objective was to establish the MTD of pevonedistat in combination with docetaxel, with carboplatin plus paclitaxel, and with gemcitabine. Secondary objectives included disease response and pharmacokinetics of pevonedistat plus standard-of-care regimens. An exploratory objective was to identify potential biomarkers of response to pevonedistat-containing therapy, including tumor ERCC1 expression.

### Assessments

Adverse events (AEs) were evaluated according to the National Cancer Institute Common Terminology Criteria for Adverse Events, version 4.03. DLT determination criteria are described in the Online Resource. Investigator-assessed tumor responses were based on Response Evaluation Criteria In Solid Tumors (RECIST), version 1.1 [26]. Response assessment methods are described in the Online Resource. Blood sampling methods for pharmacokinetic analyses, immunohistochemistry and ERCC1 expression evaluation methods, and statistical methods are reported in the Online Resource.

## Results

### Patients

At data cutoff (01 April 2016), 64 patients had been enrolled (22 in arm 1, six in arm 2a, 26 in arm 2, and 10 in arm 3) and had received at least one dose of study drug. Patient demographics and baseline characteristics were generally similar between arms (Table 1). The median number of treatment cycles was 4 (range, 1–21), and 26 (41%) patients completed ≥5 cycles (Online Resource: Supplementary Table 1). At data cutoff, 62 (97%) patients had discontinued treatment (mostly due to disease progression [*n* = 39] and AEs [*n* = 11]). Two patients remained on study in arm 2, receiving single-agent pevonedistat 20 mg/m^2^ as maintenance.

**Table 1:** Patient demographics and baseline characteristics (safety population)

	Total (*N* = 64)	Arm 1: pevonedistat + docetaxel (*n* = 22)	Arm 2a (lead-in cohort): pevonedistat + carboplatin (*n* = 6)	Arm 2: pevonedistat + carboplatin + paclitaxel (*n* = 26)	Arm 3: pevonedistat + gemcitabine (*n* = 10)
Gender					
Male / female, No. (%)	30 (47) / 34 (53)	9 (41) / 13 (59)	2 (33) / 4 (67)	13 (50) / 13 (50)	6 (60) / 4 (40)
Race					
White / Black or African American, No. (%)	52 (81) / 12 (19)	16 (73) / 6 (27)	4 (67) / 2 (33)	23 (88) / 3 (12)	9 (90) / 1 (10)
Median age, years (range)	60.5 (26–84)	61.0 (29–76)	54.5 (46–72)	61.0 (26–77)	55.5 (42–84)
Median body surface area, m^2^ (range)	1.91 (1.42–2.78)	1.89 (1.42–2.51)	1.75 (1.47–2.18)	1.96 (1.54–2.78)	1.87 (1.62–2.41)
ECOG PS 0 / 1, No. (%)	21 (33) / 43 (67)	8 (36) / 14 (64)	2 (33) / 4 (67)	7 (27) / 19 (73)	4 (40) / 6 (60)
Median time since initial diagnosis, months (range)	26.6 (2.2–126.6)	22.9 (2.2–126.6)	34.7 (18.6–55.4)	30.3 (12.9–113.4)	16.5 (4.6–60.5)
Most common (≥2 patients) disease type, No. (%)					
NSCLC, adenocarcinoma^a^	13 (20)	5 (23)	2 (33)	2 (8)	4 (40)
Breast	6 (9)	2 (9)	1 (17)	3 (12)	0 (0)
Ovarian	4 (6)	1 (5)	1 (17)	2 (8)	0 (0)
Head and neck^b^	6 (9)	2 (9)	1 (17)	3 (12)	0 (0)
NSCLC, squamous carcinoma	4 (6)	0 (0)	0 (0)	3 (12)	1 (10)
Cholangiocarcinoma	2 (3)	2 (9)	0 (0)	0 (0)	0 (0)
Colon	2 (3)	0 (0)	1 (17)	1 (4)	0 (0)
Melanoma	2 (3)	1 (5)	0 (0)	1 (4)	0 (0)
NSCLC not otherwise specified	2 (3)	2 (9)	0 (0)	0 (0)	0 (0)

Abbreviations: ECOG PS, Eastern Cooperative Oncology Group performance score; NSCLC, non-small cell lung cancer

^a^Includes 12 patients with adenocarcinoma and one with large cell adenocarcinoma

^b^Includes one patient with parotid gland carcinoma (arm 2), one with salivary gland carcinoma (arm 1), and one with squamous cell carcinoma of the oropharynx (arm 2)

### DLTs and MTD

Fifty-six (88%) patients were included in the DLT-evaluable population (Table 2). In the dose-escalation phase of arm 1, none of the three patients receiving pevonedistat 15 mg/m^2^ experienced a DLT, and two of 12 patients receiving pevonedistat 25 mg/m^2^ experienced ≥1 DLT (grade 3 increased alanine aminotransferase [ALT]/aspartate aminotransferase [AST]). Therefore, the MTD for pevonedistat was established as 25 mg/m^2^ in combination with docetaxel. Six additional patients (of whom five were DLT evaluable) were enrolled in the expansion portion at this dose to further characterize safety and tolerability (17 total DLT-evaluable patients), with two additional patients experiencing ≥1 DLT (grade 3 ALT/AST elevation).

**Table 2:** DLTs occurring during cycle 1 in the dose-escalation and MTD expansion phases and in the DLT-evaluable population

Treatment arm	Pevonedistat dose, mg/m^2^	No. of patients	No. of patients with G ≥3 DLT	DLT	Management of DLTs
Arm 1: pevonedistat + docetaxel (*n* = 20)	15	3 (dose-escalation)	0	None	NA
	25 (MTD)	12 (dose-escalation)	2	G3 increased ALT + G3 increased AST	Dose hold and dose reduction
				G3 increased ALT + G3 increased AST	Dose hold and dose reduction
		5 (MTD expansion)	2	G3 increased ALT	Dose hold and dose reduction
				G3 increased ALT + G3 increased AST	Dose hold
Arm 2a (lead-in cohort): pevonedistat + carboplatin (*n* = 6)	15	6	2	G4 thrombocytopenia	Dose delayed and concomitant medication
				G3 increased AST	Dose delayed
Arm 2: pevonedistat + carboplatin + paclitaxel (*n* = 22)	15	5 (dose-escalation)	1	G3 febrile neutropenia	Dose reduction and concomitant medication
	25	5 (dose-escalation)	2	G3 increased ALT + G3 increased AST	Dose hold and dose reduction
				G3 increased AST^a^	Dose hold and dose reduction
	20 (MTD)	6 (dose-escalation)	2	G3 increased ALT + G3 increased AST	Dose hold and dose reduction
				G3 increased ALT + G3 increased AST	Dose hold and dose reduction
		6 (MTD expansion)	1	G3 increased AST	Dose reduction
Arm 3: Pevonedistat + gemcitabine (*n* = 8)	25	8	3	G4 febrile neutropenia	Discontinued from study
				G3 febrile neutropenia + G5 febrile neutropenia	Concomitant medication, then discontinued from study
				G3 increased ALT + G3 increased AST	Dose hold and dose reduction

Abbreviations: ALT, alanine aminotransferase; AST, aspartate aminotransferase; CRM, continual reassessment method; D, day; DLT, dose-limiting toxicity; G, grade; MTD, maximum tolerated dose; NA, not applicable

^a^DLT was not considered related to study drug but was used in the CRM algorithm to de-escalate the next dose to 15 mg/m^2^

In arm 2a, two of six patients receiving pevonedistat 15 mg/m^2^ plus carboplatin AUC_6_ experienced DLTs: grade 4 thrombocytopenia and grade 3 elevated AST. Due to the toxicity observed in this lead-in cohort, patients enrolled in subsequent arm 2 cohorts received carboplatin AUC_5_ and paclitaxel 175 mg/m^2^.

In arm 2, no DLTs were observed in an initial cohort of patients treated with pevonedistat 15 mg/m^2^. Following the CRM, pevonedistat was escalated to 25 mg/m^2^, and two of five patients experienced DLTs (grade 3 increased AST and grade 3 increased ALT and AST). This led to dose de-escalation of pevonedistat (15 mg/m^2^) and, among three additional patients enrolled at this dose level, one DLT (grade 3 febrile neutropenia) was observed. Per protocol, patients were then enrolled to an intermediate dose level of pevonedistat 20 mg/m^2^, and two of six patients had DLTs (grade 3 increased ALT and AST). Based on all DLTs to date and according to the CRM algorithm, the predicted MTD at this stage was 18.1 mg/m^2^. As the predicted MTD was between the mid_low_ (17.5 mg/m^2^) and mid_high_ (22.5 mg/m^2^) (see the Online Resource) and because at least six patients had been enrolled at the 20 mg/m^2^ dose, the CRM algorithm did not suggest further dose escalation or de-escalation and the MTD was declared as 20 mg/m^2^ (Online Resource: Supplementary Fig. 1 b). Six additional patients were enrolled at this dose (expansion phase) for a total of 12 patients receiving pevonedistat at the MTD (20 mg/m^2^) in combination with paclitaxel plus carboplatin; one of these six patients reported a DLT (grade 3 increased AST), confirming the MTD in this arm.

In arm 3, eight patients enrolled at the initial dose of pevonedistat 25 mg/m^2^ were DLT evaluable and three experienced a DLT: one had grade 3 increased ALT and AST, one had grade 4 febrile neutropenia and withdrew from the study, and one had grade 3 febrile neutropenia, withdrew from the study, and died from complications of febrile neutropenia. Due to the number of DLTs observed in this arm, and based on two patients missing >1 treatment dose in cycle 1, as well as the need to delay therapy in cycle 2 to allow recovery from gemcitabine-related myelosuppression toxicity, no additional patients were enrolled in accordance with the CRM algorithm. Pevonedistat dose de-escalation to 15 mg/m^2^ was not explored, as myelosuppression was considered related to gemcitabine. The MTD of pevonedistat in combination with gemcitabine was not determined, and this drug combination was abandoned.

Of 15 patients with cycle 1 DLTs, 10 had liver function test (LFT) elevations (increased AST/ALT), which were the predominant DLTs reported across all arms regardless of standard-of-care chemotherapy. All LFT elevations were without clinical sequelae and reversible with dose delays or holds; prior therapies for these patients are reported in the Online Resource: Supplementary Table 2.

### Safety

All 64 patients experienced ≥1 AE and 53 (83%) had grade ≥3 AEs (Table 3). The most common any-grade AEs were fatigue (56%), nausea (50%), anemia (41%), constipation, diarrhea (34% each), increased AST (31%), and vomiting (30%). The most common grade ≥3 AEs were decreased neutrophil count (22%) and increased AST (20%). Overall, 95% of patients had ≥1 drug-related AE of any grade and 66% had grade ≥3 drug-related AEs (Online Resource: Supplementary Table 3). Across arms, 26 (41%) patients experienced ≥1 serious AE, including febrile neutropenia (9%), dyspnea (6%), abdominal pain, and pneumonia (3% each).

**Table 3:** Most common any-grade AEs (≥20% of all patients) and grade ≥3 AEs (≥10% of all patients) (safety population)

	Total (*N* = 64)	Arm 1: pevonedistat + docetaxel (*n* = 22)	Arm 2a (lead-in cohort): pevonedistat + carboplatin (*n* = 6)	Arm 2: pevonedistat + carboplatin + paclitaxel (*n* = 26)	Arm 3: pevonedistat + gemcitabine (*n* = 10)
Patients with one or more any-grade AE, *n* (%)	64 (100)	22 (100)	6 (100)	26 (100)	10 (100)
Fatigue	36 (56)	12 (55)	4 (67)	16 (62)	4 (40)
Nausea	32 (50)	8 (36)	5 (83)	14 (54)	5 (50)
Anemia	26 (41)	7 (32)	3 (50)	12 (46)	4 (40)
Constipation	22 (34)	4 (18)	4 (67)	11 (42)	3 (30)
Diarrhea	22 (34)	9 (41)	1 (17)	11 (42)	1 (10)
Increased AST	20 (31)	6 (27)	2 (33)	9 (35)	3 (30)
Vomiting	19 (30)	4 (18)	1 (17)	11 (42)	3 (30)
Increased ALT	18 (28)	7 (32)	1 (17)	6 (23)	4 (40)
Alopecia	17 (27)	5 (23)	1 (17)	10 (38)	1 (10)
Decreased appetite	15 (23)	4 (18)	0 (0)	6 (23)	5 (50)
Decreased neutrophil count	15 (23)	6 (27)	1 (17)	7 (27)	1 (10)
Thrombocytopenia	15 (23)	2 (9)	3 (50)	7 (27)	3 (30)
Dyspnea	14 (22)	6 (27)	2 (33)	3 (12)	3 (30)
Neutropenia	14 (22)	2 (9)	1 (17)	9 (35)	2 (20)
Peripheral sensory neuropathy	14 (22)	3 (14)	1 (17)	9 (35)	1 (10)
Pyrexia	14 (22)	6 (27)	1 (17)	5 (19)	2 (20)
Arthralgia	13 (20)	2 (9)	0 (0)	9 (35)	2 (20)
Myalgia	13 (20)	3 (14)	0 (0)	10 (38)	0
Decreased platelet count	13 (20)	1 (5)	2 (33)	7 (27)	3 (30)
Patients with one or more grade ≥3 AE, *n* (%)	53 (83)	18 (82)	4 (67)	21 (81)	10 (100)
Decreased neutrophil count	14 (22)	6 (27)	0 (0)	7 (27)	1 (10)
Increased AST	13 (20)	4 (18)	2 (33)	6 (23)	1 (10)
Anemia	12 (19)	3 (14)	2 (33)	6 (23)	1 (10)
Increased ALT	11 (17)	5 (23)	0 (0)	4 (15)	2 (20)
Neutropenia	10 (16)	1 (5)	1 (17)	7 (27)	1 (10)
Thrombocytopenia	7 (11)	0 (0)	2 (33)	4 (15)	1 (10)

Abbreviations: AE, adverse event; ALT, alanine aminotransferase; AST, aspartate aminotransferase

Four patients (arm 2) reported AEs leading to permanent treatment discontinuation. Of these, one patient receiving pevonedistat 15 mg/m^2^ in combination with carboplatin plus paclitaxel discontinued due to thrombocytopenia, and three patients (two receiving pevonedistat 20 mg/m^2^ and one 25 mg/m^2^, both with carboplatin plus paclitaxel) discontinued due to peripheral neuropathy or peripheral sensory neuropathy.

There were five on-study deaths, including one on day 23, cycle 1 that was considered drug-related (arm 3, pevonedistat 25 mg/m^2^: febrile neutropenia). A further three patients died during cycle 1 and one died during cycle 9, all of which were considered unrelated to treatment (all pevonedistat 25 mg/m^2^; one each in arm 1 and arm 2, two in arm 3).

### Pevonedistat pharmacokinetics

**Fig. 1:**
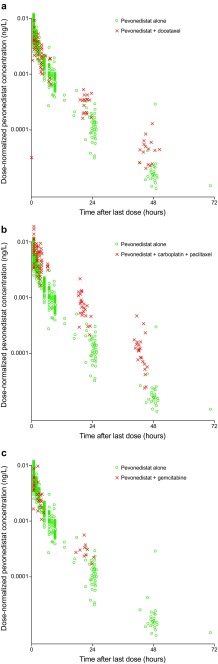
Observed dose-normalized concentration-time profiles of pevonedistat with and without concurrent administration of docetaxel (**a**), carboplatin plus paclitaxel (**b**), or gemcitabine (**c**). The figure shows raw concentration-time data from prior studies of single-agent pevonedistat (circles) [7–9, 11] and from the combination therapy in this study (crosses)

Figure 1 compares the dose-normalized pevonedistat concentrations for patients receiving pevonedistat in combination with docetaxel (*n* = 16, dose-escalation; *n* = 6, MTD expansion), carboplatin plus paclitaxel (*n* = 20 dose-escalation; *n* = 6, MTD expansion), or gemcitabine (*n* = 10) with those from patients who received single-agent pevonedistat in previous studies [7–9, 11]. Examination of individual patient plasma concentration-time data (Fig. 1a and c) revealed no significant changes in pevonedistat pharmacokinetics when given with docetaxel (arm 1) or gemcitabine (arm 3), as the observed pevonedistat concentrations in the presence of docetaxel or gemcitabine showed considerable overlap with those with pevonedistat alone. By contrast, following the end of infusion and throughout the 48-h sampling period, pevonedistat plasma concentrations were consistently higher in patients receiving carboplatin plus paclitaxel than in patients receiving single-agent pevonedistat (Fig. 1b).

### Disease response

A summary of best response to treatment is shown in Table 4. In arm 1, among 19 response-evaluable patients, three patients receiving the MTD achieved a partial response (PR), for an overall response rate (ORR) of 16%. Among responders, one had cholangiocarcinoma, two had head and neck cancer (squamous cell carcinoma and salivary gland carcinoma), and all three had lung metastases. One patient in arm 2a (squamous cell carcinoma of the head and neck not otherwise specified) achieved a PR.

**Table 4:** Summary of best response to treatment (response-evaluable population)

	Total (*N* = 54)	Arm 1: pevonedistat + docetaxel (*n* = 19)	Arm 2a (lead-in cohort): pevonedistat + carboplatin (*n* = 6)	Arm 2: pevonedistat + carboplatin + paclitaxel (*n* = 23)	Arm 3: pevonedistat + gemcitabine (*n* = 6)
ORR (CR + PR), *n* (%)	12 (22)	3 (16)	1 (17)	8 (35)	0 (0)
CR	2 (4)^a^	0 (0)	0 (0)	2 (9)	0 (0)
PR	10 (19)^b^	3 (16)	1 (17)	6 (26)	0 (0)
SD, *n* (%)	29 (54)	9 (47)	3 (50)	12 (52)	5 (83)
PD, *n* (%)	13 (24)	7 (37)	2 (33)	3 (13)	1 (17)

Abbreviations: CR, complete response; ORR, overall response rate; PD, progressive disease; PR, partial response; SD, stable disease

^a^Tumor types: bladder carcinoma (patient received prior platinum therapy) and endometrial cancer (patient received both platinum and taxane as prior therapy)

^b^Tumor types: biphasic hepatocellular carcinoma and cholangiocarcinoma, breast cancer, cholangiocarcinoma, follicular dendritic cell sarcoma, salivary gland carcinoma, squamous cell carcinoma of the head and neck not otherwise specified, squamous cell carcinoma of the oropharynx, squamous non-small cell lung cancer, and parotid gland carcinoma

In arm 2, among 23 response-evaluable patients, eight patients achieved a PR or complete response (CR; ORR 35%). Two patients with bladder cancer and endometrial cancer receiving pevonedistat 20 mg/m^2^ (MTD) achieved a CR; at data cutoff, the duration of CR was 8.1 and 8.5 months (9 and 11 cycles), respectively. At the time of reporting, both patients remained on study (cycle 43+ representing the longest treatment duration) receiving single-agent pevonedistat since cycle 9 and 13, respectively. Of the six patients achieving PRs in arm 2, two received pevonedistat 25 mg/m^2^ and four received pevonedistat 20 mg/m^2^; all six had progressive disease after PR.

Median duration of response (DOR) for the 12 patients achieving a CR or PR across both arms was 5.9 months (range, 0.03–12.02). Median DOR for the eight patients receiving pevonedistat with carboplatin and paclitaxel (arm 2) was 7.4 months (range, 2.37–12.02). Across 54 response-evaluable patients, 41 achieved stable disease or better (≥SD), of whom 25 received treatment for ≥5 cycles (eight in arm 1, 15 in arm 2, and two in arm 3), with the longest treatment duration being 21 cycles at data cutoff (arm 2, chondrosarcoma). At the pevonedistat MTDs, 10 of 16 patients in arm 1 (25 mg/m^2^) and all 12 patients in arm 2 (20 mg/m^2^) achieved ≥SD; of these, six and nine patients, respectively, received study treatment for ≥5 cycles.

Prior therapies in patients achieving a response are presented in the Online Resource: Supplementary Table 4. Of 12 patients achieving a CR or PR, 10 had received prior platinum therapy. None of the responders in arm 1 (pevonedistat plus docetaxel) had received prior docetaxel, and one patient had received prior carboplatin plus paclitaxel. By contrast, all but one patient achieving a CR or PR in arm 2 (pevonedistat in combination with carboplatin and paclitaxel) and one responder in arm 2a (pevonedistat plus carboplatin) had received ≥1 course of platinum and/or taxane. In arm 2, four patients had received both taxane and platinum as prior therapies.

### ERCC1 expression

**Fig. 2:**
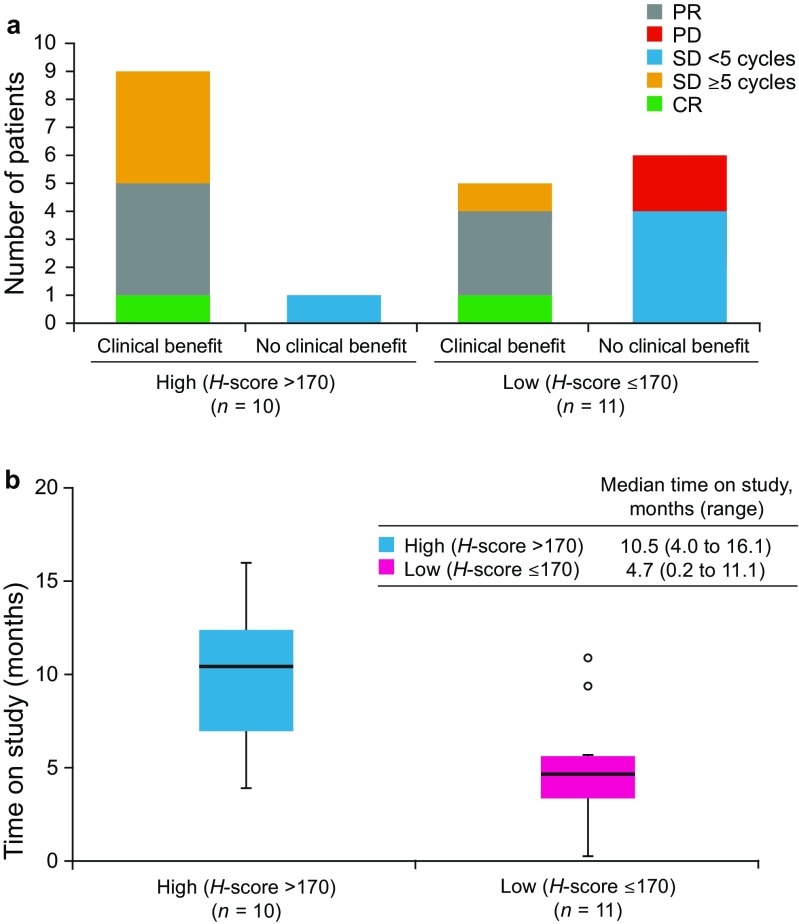
Correlation between baseline ERCC1 expression levels* and (**a**) clinical benefit (CR/PR/durable SD ≥5 cycles) or (**b**) time on study. (**a**) *ERCC1 staining intensity was determined by immunohistochemistry and the *H*-score was calculated. The median *H*-score of 170 for the ERCC1-evaluable patient population (*n* = 21) was used as the cutoff to dichotomize patients into ERCC1 high (*H-*score > 170, *n* = 10) and low (*H*-score ≤170, *n* = 11) expression level groups. (**b**) The black horizontal lines inside the boxes represent the median time on study for the ERCC1 high and low expression level groups. Time on study (months) was defined as follows: (last study visit date – first dose date +1)/30.4375. This was 10.5 and 4.7 months for patients with ERCC1 high and low expression levels, respectively. Box plots represent interquartile range. Outliers are indicated by the highlighted circles. CR, complete response; ERCC1, Excision repair cross-complementation group 1; PD, progressive disease; PR, partial response; SD, stable disease

ERCC1 expression levels were determined by calculating the *H*-score in patients receiving pevonedistat 20 mg/m^2^ or 15 mg/m^2^ in arm 2 and arm 2a. The median *H*-score for all ERCC1-evaluable patients (*n* = 21) was 170 (range, 65–290), which served as the cutoff to classify patients into ERCC1 high (*H*-score > 170, *n* = 10) and low (*H*-score ≤170, *n* = 11) expression level groups. High ERCC1 expression appeared to correlate with clinical benefit (CR/PR/SD duration ≥5 cycles; Fig. 2a). There was a trend for patients with high ERCC1 levels to remain on study longer than patients with low ERCC1, with a median time on study of 10.5 and 4.7 months, respectively (Fig. 2b).

## Discussion

This phase Ib study was the first to investigate the safety and tolerability of pevonedistat plus standard-of-care chemotherapies in patients with advanced solid tumors. The MTD for pevonedistat was determined to be 25 mg/m^2^ with docetaxel and 20 mg/m^2^ with carboplatin plus paclitaxel. The combination of pevonedistat plus gemcitabine was deemed intolerable due to two patient withdrawals and one on-study death (febrile neutropenia), and based on patients missing ≥1 treatment dose (cycle 1) or experiencing gemcitabine-related myelosuppression which led to dose delays in cycle 2. Therefore, enrollment into this cohort ceased. The most frequently reported DLTs across all arms included grade 3 febrile neutropenia and increased ALT/AST; AST/ALT elevations were reversible with dose holds, reductions, or delays.

The safety profile of pevonedistat plus docetaxel or carboplatin plus paclitaxel was generally favorable. Common AEs reported in this study (fatigue, nausea, anemia, constipation, and diarrhea) were similar to those previously observed in patients receiving standard-of-care chemotherapies [27–29]. With dose reductions or delays, repeat dosing of pevonedistat plus docetaxel or pevonedistat in combination with carboplatin plus paclitaxel was feasible in patients with solid tumors, and at the time of reporting, two patients (arm 2) remained on study receiving single-agent pevonedistat 20 mg/m^2^ (currently cycle 43+). Pevonedistat treatment did not appear to result in additional toxicity when added to standard-of-care chemotherapy, and there was no cumulative toxicity from pevonedistat despite prolonged treatment.

Evaluation of concentration-time data from 58 patients for whom blood samples were available revealed that the pharmacokinetic profile of pevonedistat was unchanged in the presence of docetaxel or gemcitabine compared with historical single-agent pevonedistat pharmacokinetic data [7–9, 11]. There was a trend toward higher pevonedistat plasma concentrations when given with carboplatin plus paclitaxel, compared with single-agent administration. This apparent drug-drug interaction effect, which cannot be explained at this time, warrants further investigation. Although some overlap exists in the metabolizing enzymes and/or transporter proteins involved in the drug clearance of pevonedistat and the standard-of-care agents, none are known inhibitors or inducers. A radiolabeled mass balance study is ongoing to characterize the clearance pathways of pevonedistat in humans (ClinicalTrials.gov identifier NCT03057366). Additionally, because of the limited pharmacokinetic sampling scheme used in this study, individual concentration-time data will be pooled with other study data and analyzed using a population modeling approach to quantify the observed effects of carboplatin and paclitaxel on pevonedistat pharmacokinetics.

The combination of pevonedistat with carboplatin plus paclitaxel (arm 2) showed the most promising broad-based antitumor activity in pretreated patients (≥1 prior therapies). Notably, all but one of the eight responders in arm 2 and one responder in arm 2a had previously received platinum, taxanes, or both. The ORR in arm 2 was 35%, including two patients with CR (bladder cancer and endometrial carcinoma). These two patients discontinued carboplatin plus paclitaxel chemotherapy at cycle 9 and 13, respectively, and went on to maintenance therapy with single-agent pevonedistat; both patients are currently continuing treatment in cycle 43 without evidence of disease. Consistent with preclinical studies reporting synergy between pevonedistat and standard-of-care chemotherapy [13, 24], the long treatment durations in this study and objective responses in patients resistant to prior platinum/taxane therapy suggest the potential reversal of resistance by the addition of pevonedistat. Further exploration of pevonedistat and carboplatin plus paclitaxel in the platinum- and/or taxane-resistant setting is warranted.

Pevonedistat was shown in model systems to synergize with platinum-containing agents by interfering with NER and ICR pathways [13]. Previous studies reported that low ERCC1 expression is associated with clinical benefit in advanced cancer patients receiving platinum-based chemotherapy [19–21]. Interestingly, in our study, patients receiving pevonedistat plus paclitaxel and carboplatin, who had high ERCC1 expression appeared to show greater clinical benefit and remained on study longer than those with low ERCC1 expression. Further investigations are warranted to provide insight into this observation, which suggests the possibility that pevonedistat might re-sensitize patients to platinum-based chemotherapy and provide a potential new approach to therapy in these patients.

The observed clinical benefit in patients treated in arm 2, especially in patients with prior taxane and/or platinum exposure, supports further investigation of pevonedistat with carboplatin plus paclitaxel in phase II trials.

## Electronic supplementary material

ESM 1 (DOCX 45 kb)

Supplementary Fig. 1 (PDF 1335 kb)
