# Supplementary material for: Phase Ib study of pevonedistat, a NEDD8-activating enzyme inhibitor, in combination with docetaxel, carboplatin and paclitaxel, or gemcitabine, in patients with advanced solid tumors
Source: Invest New Drugs. 2018 May 21;37(1):87–97. doi: 10.1007/s10637-018-0610-0 (PMC6510847; doi:10.1007/s10637-018-0610-0)
Supplement: Supplementary file 1 — (DOCX 45 kb) [file 10637_2018_610_MOESM1_ESM.docx]

**Title:** Phase Ib study of pevonedistat, a NEDD8-activating enzyme inhibitor, in combination with docetaxel, carboplatin and paclitaxel, or gemcitabine, in patients with advanced solid tumors

**Journal:** Investigational New Drugs

**Authors**: A. Craig Lockhart^1^ • Todd M. Bauer^2^ • Charu Aggarwal^3^ • Carrie B. Lee^4^ • R. Donald Harvey^5^ • Roger B. Cohen^6^ • Farhad Sedarati^7^ • Tsz Keung Nip^8^ • Hélène Faessel^9^ • Ajeeta B. Dash^10^ • Bruce J. Dezube^7^ • Douglas V. Faller^7^ • Afshin Dowlati^11^

**Corresponding author:** A. Craig Lockhart, MD, Division of Medical Oncology, University of Miami/Sylvester Comprehensive Cancer Center 1120 NW 14^th^ Street, Suite 650L, Miami, FL 33136; Telephone: 305-243-0116; Email: [aclockhart@med.miami.edu](mailto:alockhar@DOM.wustl.edu); Fax: 305-243-9161

**Online resource – Online only**

**Methods**

**Study design and patients**

Eligible patients were aged ≥18 years and had histologically or cytologically confirmed metastatic or locally advanced and incurable solid tumors that could benefit from standard-of-care therapy (docetaxel, carboplatin plus paclitaxel, or gemcitabine), or that had progressed despite standard therapy, or for which conventional therapy was not considered effective. Furthermore, patients were required to have an Eastern Cooperative Oncology Group performance status of 0 or 1; recovered from previous antineoplastic therapy (grade ≤1 toxicity); and adequate hematologic, hepatic, and renal functions. Exclusion criteria were systemic antineoplastic therapy or treatment with other investigational products within 21 days before the first dose of pevonedistat; radiotherapy, surgery, or treatment with cytochrome P450 3A inducers/inhibitors within 14 days before the first dose of pevonedistat; prior treatment with radiation therapy involving ≥25 % of the hematopoietically active bone marrow; or a known hypersensitivity to any components of the treatment regimens.

**Treatments**

All study drugs were administered intravenously. In arm 1, pevonedistat and docetaxel were each administered as 60-minute infusions; on days when both drugs were administered, docetaxel was administered first. Patients in arm 2 received pevonedistat, carboplatin, and paclitaxel as 60-, 30-, and 180-minute infusions, respectively; when all three drugs were administered, paclitaxel was administered first, followed by carboplatin, and then pevonedistat. In arm 3, patients received pevonedistat and gemcitabine over 60 minutes, with gemcitabine being administered first. In all three arms, on the days that pevonedistat was administered with another drug, a time-out period of ~15 minutes was required between the end of chemotherapy and start of pevonedistat infusion.

**Dose-limiting toxicities (DLTs)**

A DLT was defined as any of the following considered related to any study drug: any grade 4 hematologic toxicity, except for grade 4 neutropenia lasting <7 days; grade ≥3 neutropenia with fever >38.5ºC lasting ≥1 hour; grade ≥3 non-hematologic toxicity except for brief (<1 week) fatigue or hypophosphatemia; grade ≥2 non-hematologic toxicity requiring pevonedistat dose reduction/discontinuation; a delay of >4 weeks starting cycle 2 due to a hematologic toxicity not related to tumor infiltration (bone marrow evaluation may be required), or delay of >2 weeks due to non-hematologic toxicity; any adverse event during cycle 1 leading to dose modification in the next cycle; or any drug-related toxicity resulting in administration of only one dose of gemcitabine per cycle. In arm 3, based on the myelosuppression risk associated with gemcitabine treatment, one dose hold of gemcitabine was allowed and did not constitute a DLT.

**Dose-escalation rules and MTD determination**

An adaptive approach using a continual reassessment method (CRM) was used for dose escalation to model the relationship between toxicities and dose level and to determine the maximum tolerated dose (MTD) for arms 1, 2, and 3 (Online Resource: Supplementary Fig. 1b). Using the CRM, the dose-toxicity relationship and the predicted MTD (PMTD) level were updated based on the observed DLTs after patients completed the first cycle of therapy. The recommendation to escalate, de-escalate, or expand at the same dose level was determined by comparing the PMTD level from the dose-toxicity model to the pre-specified dose levels.

Three patients were dosed at the initial dose level and, during treatment at the subsequent dose levels, dose escalation depended upon the observed DLT rate in all previously treated patients. For safety reasons, no dose level was skipped and a minimum of three patients at the current dose level was required before escalating to the next higher dose level. To decide whether to enroll additional patients and hold, escalate, or de-escalate the dose, the midpoint between the current and next higher dose level (mid_high_) and the midpoint between the previous and current dose level (mid_low_) was calculated (Online Resource: Supplementary Fig. 1b). After at least six patients were treated at any dose level and the algorithm did not recommend escalation or de-escalation, this dose level was considered the MTD of pevonedistat in combination with standard-of-care chemotherapy. After determining the MTD of pevonedistat, approximately six additional patients were to be enrolled into each arm (total of ~12 patients treated at the MTD) to characterize the safety, tolerability, and pharmacokinetics of pevonedistat with chemotherapy.

**Assessments**

Tumor response was assessed by the investigators using the Response Evaluation Criteria In Solid Tumors (RECIST), version 1.1, which considers target and non-target tumor status [1]. Computed tomography (CT) scans with intravenous contrast of the chest, abdomen, and pelvis were performed during screening, at the end of cycle 2, at the end of every other subsequent cycle, and at the end-of-study visit. If a CT scan did not provide adequate imaging, magnetic resonance imaging (MRI) scans were used to evaluate the sites of disease. If the patient had appropriate imaging scans performed within 28 days of day 1, cycle 1, then the results of those scans could be used for the screening assessment. For each site of disease, the imaging modality (CT or MRI) used at screening was to be used throughout the study.

**Pevonedistat pharmacokinetics**

To assess possible effects of standard-of-care agents on pevonedistat pharmacokinetics during treatment, blood samples were collected on day 1, cycle 1 pre-dose, at the end of infusion, and at 1.5, 3, and 20 hours post‑infusion (and at 48 hours post-dose for arms 1 and 2 only) during dose escalation. During MTD expansion (arms 1 and 2), blood samples were collected on day 1 and day 5 of cycle 1 (pre-dose, end of infusion, at 1.5, 3, 6, or 7 hours post-infusion, and at 20 and 48 hours post-dose). Plasma samples were analyzed for pevonedistat concentrations by Covance (West Trenton, NJ, USA) using liquid chromatography with tandem mass spectrometry (dynamic range: 1–500 ng/mL).

**Immunohistochemistry and excision repair cross-complementation group 1 (ERCC1) expression evaluation**

Paraffin-embedded tumor tissue blocks or a minimum of 10 unstained slides of tumor tissue were collected at screening to evaluate ERCC1 expression by immunohistochemistry (clone 4F9; Origene, Rockville, MD, USA) [2]. ERCC1 nuclear staining intensity was determined by calculating the *H*-score, using a semi‑quantitative scoring system [2]. Tumor nuclear staining intensity was graded relative to the intensity of internal stromal fibroblasts and/or endothelial cells, as well as staining of the negative control antibody. Internal positive controls, immediately adjacent to the tumor area, had to show positive staining for samples to be scored. Staining in the stromal cells was considered 2+, and the staining intensity of the tumor cells was scored in comparison to the level of 2+ exhibited in those adjacent stromal cells. Samples were scored as “not evaluable” if adjacent stromal cells showed negative staining, regardless of tumor cell staining detected in the sample. The *H*-score was calculated as follows: (3 x % tumor cells staining at 3+) + (2 x % tumor cells staining at 2+) + (1 x % tumor cells staining at 1+), giving a range of 0-300. The median *H*‑score of the ERCC1‑evaluable patient population was used as the cutoff to dichotomize patients into ERCC1 high (*H*‑score >median) and low (*H*‑score ≤median) expression level groups [2].

**Statistics and study populations**

Analyses were descriptive and exploratory in nature. The safety population was defined as all patients receiving ≥1 dose of study drug. The DLT-evaluable population was defined as all patients who either experienced a DLT during treatment cycle 1 or received all scheduled doses of study drug on scheduled days during cycle 1 without DLT. Patients in arm 3 were considered DLT-evaluable if they received all doses of pevonedistat and received gemcitabine on either days 1 and 8 or days 1 and 15 of cycle 1. The response-evaluable population was defined as all patients who received ≥1 dose of pevonedistat, had measurable disease at baseline, and had ≥1 post-baseline disease assessment.

**References**

1. Eisenhauer EA, Therasse P, Bogaerts J, et al. New response evaluation criteria in solid tumours: revised RECIST guideline (version 1.1). Eur J Cancer 2009;**45**:228–47.
2. Bahamon BN, Gao F, Danaee H. et al. Development and validation of an ERCC1 immunohistochemistry assay for solid tumors. Arch Pathol Lab Med 2016;**140**:1397–403.

**Tables**

**Supplementary Table 1** Extent of pevonedistat exposure (safety population) [online only]

|  | Total  (*N* = 64) | Arm 1:  Pevonedistat + Docetaxel  (*n* = 22) | Arm 2a  (lead-in cohort):  Pevonedistat + Carboplatin  (*n* = 6) | Arm 2:  Pevonedistat + Carboplatin + Paclitaxel  (*n* = 26) | Arm 3:  Pevonedistat + Gemcitabine  (*n* = 10) |
| --- | --- | --- | --- | --- | --- |
| Median no. of treatment cycles, *n* (range) | 4.0 (1.0–21.0) | 3.5 (1.0–10.0) | 4.0 (2.0–5.0) | 6.0 (1.0–21.0) | 2.0 (1.0–9.0) |
| Median no. of doses per cycle, *n* (range) | 2.9 (1.0–3.0) | 3.0 (2.0–3.0) | 3.0 (2.0–3.0) | 2.9 (1.0–3.0) | 2.4 (1.0–3.0) |
| No. of treated cycles, *n* (%) |  |  |  |  |  |
| ≥1 cycle | 64 (100) | 22 (100) | 6 (100) | 26 (100) | 10 (100) |
| ≥2 cycles | 52 (81) | 18 (82) | 6 (100) | 22 (85) | 6 (60) |
| ≥3 cycles | 40 (63) | 12 (55) | 4 (67) | 20 (77) | 4 (40) |
| ≥4 cycles | 38 (59) | 11 (50) | 4 (67) | 19 (73) | 4 (40) |
| ≥5 cycles | 26 (41) | 8 (36) | 1 (17) | 15 (58) | 2 (20) |
| ≥6 cycles | 24 (38) | 7 (32) | 0 (0) | 15 (58) | 2 (20) |
| ≥7 cycles | 17 (27) | 4 (18) | 0 (0) | 12 (46) | 1 (10) |
| ≥8 cycles | 14 (22) | 3 (14) | 0 (0) | 10 (38) | 1 (10) |
| ≥9 cycles | 12 (19) | 2 (9) | 0 (0) | 9 (35) | 1 (10) |
| ≥10 cycles | 11 (17) | 2 (9) | 0 (0) | 9 (35) | 0 (0) |

**Supplementary Table 2** Prior therapies in patients reporting LFT elevations [online only]

| Treatment arm | Pevonedistat dose (mg/m^2^) | Tumor type | DLT date (cycle/day) | Days from first dose | Days from last dose | Prior therapy |
| --- | --- | --- | --- | --- | --- | --- |
| Arm 1 (pevonedistat + docetaxel) | 25 | Ovarian cancer | C1D2 | 2 | 2 | Carboplatin + paclitaxel |
|  | 25 | Breast cancer | C1D2 | 2 | 2 | Carboplatin + paclitaxel + gemcitabine |
|  | 25 | Cholangiocarcinoma | C1D2 | 2 | 2 | Gemcitabine + oxaliplatin |
|  | 25 | Melanoma | C1D2 | 2 | 2 | Ifosfamide + doxorubicin, dacarbazine, ifosfamide + etoposide, irinotecan + temozolomide, vincristine + doxorubicin + cyclophosphamide |
| Arm 2a (pevonedistat + carboplatin) | 15 | Breast cancer | C2D3 | 24 | 3 | Cyclophosphamide + docetaxel + doxorubicin, capecitabine |
|  | 15 | Ovarian cancer | C1D2 | 2 | 2 | Carboplatin + paclitaxel, topotecan, doxorubicin |
| Arm 2 (pevonedistat + carboplatin  + paclitaxel) | 20 | Ovarian | C1D2 | 2 | 2 | Carboplatin + paclitaxel, topotecan, doxorubicin |
|  | 20 | Hepatocellular carcinoma, cholangiocarcinoma | C1D2 | 2 | 2 | Gemcitabine + oxaliplatin,  5-fluorodeoxyuridine |
|  | 20 | Breast cancer | C1D2 | 2 | 2 | Paclitaxel, cyclophosphamide + doxorubicin, capecitabine, gemcitabine, ixabepilone (etoposide), crizotinib |
|  | 25 | Parotid cancer | C1D5 | 5 | 3 | Carboplatin + paclitaxel, gemcitabine + carboplatin |
|  | 25 | Cervical cancer | C1D2 | 2 | 2 | Carboplatin, topotecan + cisplatin |
| Arm 3 (pevonedistat + gemcitabine) | 25 | Sarcoma | C1D2 | 2 | 2 | Sunitinib |

**Supplementary Table 3** Most common any-grade (≥10 % of patients) and grade ≥3 (≥10 % of patients) drug-related AEs (safety population) [online only]

|  | Total  (*N* = 64) | Arm 1:  pevonedistat + docetaxel  (*n* = 22) | Arm 2a  (lead-in cohort):  pevonedistat + carboplatin  (*n* = 6) | Arm 2:  pevonedistat + carboplatin + paclitaxel  (*n* = 26) | Arm 3:  pevonedistat + gemcitabine  (*n* = 10) |
| --- | --- | --- | --- | --- | --- |
| Patients with one or more any-grade drug‑related AEs, *n* (%) | 61 (95) | 22 (100) | 6 (100) | 24 (92) | 9 (90) |
| Fatigue | 30 (47) | 10 (45) | 3 (50) | 14 (54) | 3 (30) |
| Nausea | 25 (39) | 8 (36) | 3 (50) | 11 (42) | 3 (30) |
| Anemia | 21 (33) | 5 (23) | 2 (33) | 11 (42) | 3 (30) |
| Increased AST | 19 (30) | 6 (27) | 2 (33) | 9 (35) | 2 (20) |
| Increased ALT | 17 (27) | 7 (32) | 1 (17) | 6 (23) | 3 (30) |
| Diarrhea | 15 (23) | 7 (32) | 0 (0) | 7 (27) | 1 (10) |
| Decreased neutrophil count | 15 (23) | 6 (27) | 1 (17) | 7 (27) | 1 (10) |
| Alopecia | 15 (23) | 5 (23) | 1 (17) | 9 (35) | 0 (0) |
| Neutropenia | 14 (22) | 2 (9) | 1 (17) | 9 (35) | 2 (20) |
| Thrombocytopenia | 14 (22) | 1 (5) | 3 (50) | 7 (27) | 3 (30) |
| Decreased appetite | 14 (22) | 3 (14) | 0 (0) | 6 (23) | 5 (50) |
| Sensory peripheral neuropathy | 14 (22) | 3 (14) | 1 (17) | 9 (35) | 1 (10) |
| Vomiting | 13 (20) | 3 (14) | 1 (17) | 8 (31) | 1 (10) |
| Decreased platelet count | 13 (20) | 1 (5) | 2 (33) | 7 (27) | 3 (30) |
| Myalgia | 10 (16) | 2 (9) | 0 (0) | 8 (31) | 0 (0) |
| Flushing | 10 (16) | 4 (18) | 2 (33) | 4 (15) | 0 (0) |
| Peripheral neuropathy | 9 (14) | 1 (5) | 0 (0) | 7 (27) | 1 (10) |
| Dysgeusia | 8 (13) | 2 (9) | 3 (50) | 2 (8) | 1 (10) |
| Constipation | 7 (11) | 2 (9) | 2 (33) | 3 (12) | 0 (0) |
| Decreased WBC count | 7 (11) | 3 (14) | 0 (0) | 4 (15) | 0 (0) |
| Arthralgia | 7 (11) | 1 (5) | 0 (0) | 5 (19) | 1 (10) |
| Patients with one or more grade ≥3 drug‑related AEs, *n* (%) | 42 (66) | 14 (64) | 3 (50) | 17 (65) | 8 (80) |
| Decreased neutrophil count | 14 (22) | 6 (27) | 0 (0) | 7 (27) | 1 (10) |
| Increased AST | 12 (19) | 4 (18) | 2 (33) | 5 (19) | 1 (10) |
| Increased ALT | 11 (17) | 5 (23) | 0 (0) | 4 (15) | 2 ( 20) |
| Neutropenia | 10 (16) | 1 (5) | 1 (17) | 7 (27) | 1 (10) |
| Anemia | 9 (14) | 2 (9) | 2 (33) | 5 (19) | 0 (0) |
| Thrombocytopenia | 7 (11) | 0 (0) | 2 (33) | 4 (15) | 1 (10) |

Abbreviations: AE, adverse event; ALT, alanine aminotransferase; AST, aspartate aminotransferase; WBC, white blood cell.

**Supplementary Table 4** Prior therapies in patients achieving CR and PR [online only]

| Regimen | Tumor type | Number of cycles received | Response | Prior therapies (best response, if known) |
| --- | --- | --- | --- | --- |
| Pevonedistat 20 mg/m^2^ + carboplatin + paclitaxel | Bladder carcinoma | 15^a^ | CR | Carboplatin + gemcitabine (PD); cisplatin + gemcitabine (SD); ipilimumab + nivolumab |
| Pevonedistat 20 mg/m^2^ + carboplatin + paclitaxel | Endometrial cancer | 12^a^ | CR | Taxol + doxorubicin + cisplatin (SD); surgery; radiation |
| Pevonedistat 25 mg/m^2^ + docetaxel | Head and neck cancer: salivary gland carcinoma, with head and neck, lung, and lymph nodes involvement | 8 | PR | Carboplatin + paclitaxel (PD); surgery; radiation |
| Pevonedistat 25 mg/m^2^ + docetaxel | Head and neck squamous cell carcinoma | 10 | PR | Cisplatin (CR), carboplatin + 5FU + cetuximab (SD); surgery (SD); radiation |
| Pevonedistat 25 mg/m^2^ + docetaxel | Cholangiocarcinoma | 3 | PR | Gemcitabine; fluorouracil; cisplatin + gemcitabine (PD);  fluorouracil + leucovorin; gemcitabine + cetuximab + oxaliplatin; surgery (PD); radiation |
| Pevonedistat 15 mg/m^2^ + carboplatin | Squamous cell carcinoma of the head and neck not otherwise specified | 3 | PR | Cetuximab; cetuximab + paclitaxel;  carboplatin + fluorouracil + leucovorin;  romidepsin; radiation |
| Pevonedistat 20 mg/m^2^ + carboplatin + paclitaxel | Breast cancer | 12 | PR | Bevacizumab + paclitaxel; cyclophosphamide + doxorubicin; capecitabine; gemcitabine; ixabepilone; eribulin; crizotinib; surgery; radiation |
| Pevonedistat 20 mg/m^2^ + carboplatin + paclitaxel | Head and neck cancer, parotid gland carcinoma | 12 | PR | Carboplatin + paclitaxel (SD); belinostat; surgery; radiation |
| Pevonedistat 20 mg/m^2^ + carboplatin + paclitaxel | Squamous cell carcinoma of the oropharynx | 6 | PR | Bevacizumab + docetaxel + carboplatin;  nivolumab; cetuximab; methotrexate |
| Pevonedistat 20 mg/m^2^ + carboplatin + paclitaxel | Biphasic hepatocellular carcinoma and cholangiocarcinoma | 8 | PR | Floxuridine; gemcitabine + oxaliplatin; surgery; radiation |
| Pevonedistat 25 mg/m^2^ + carboplatin + paclitaxel | Squamous non-small cell lung carcinoma | 12 | PR | Carboplatin + paclitaxel; docetaxel; gemcitabine; surgery; radiation |
| Pevonedistat 25 mg/m^2^ + carboplatin + paclitaxel | Follicular dendritic cell sarcoma | 18 | PR | Surgery |

Abbreviations: 5FU, 5-fluorouracil; CR, complete response; PD, progressive disease; PR, partial response; SD, stable disease.

^a^Number of cycles received at data cutoff date; at time of reporting patient is currently active on study (cycle 43+) without evidence of disease.

**Figure**

**Supplementary Fig. 1** (a) Study design and (b) dose-escalation scheme.

^a^Patients in the lead-in cohort of arm 2 (arm 2a) did not enroll in the dose-escalation phase.

^b^Planned dose levels of pevonedistat. Evaluation of intermediate doses was permitted as per protocol.

^c^The planned doses of paclitaxel 200 mg/m^2^ and carboplatin AUC_6_ were reduced to 175 mg/m^2^ and AUC_5_, respectively, in arm 2 due to toxicity observed in the lead-in cohort (arm 2a).

^d^The intervals between dose levels are pre-specified and not determined by the CRM algorithm.

AUC_5_, area under the curve concentration-time curve (AUC_5_) mg/mL·min; AUC_6_, AUC 6 mg/mL·min; CRM, continual reassessment method; DLT, dose-limiting toxicity; IV, intravenous; Mid_high_, midpoint between current dose level and next dose level; Mid_low_, midpoint between previous dose level and current dose level; MTD, maximum tolerated dose; PMTD, predicted MTD. [Online only]
