# Supplementary figures and images for: Phase Ib study of pevonedistat, a NEDD8-activating enzyme inhibitor, in combination with docetaxel, carboplatin and paclitaxel, or gemcitabine, in patients with advanced solid tumors
Source: Invest New Drugs. 2018 May 21;37(1):87–97. doi: 10.1007/s10637-018-0610-0 (PMC6510847; doi:10.1007/s10637-018-0610-0)

a

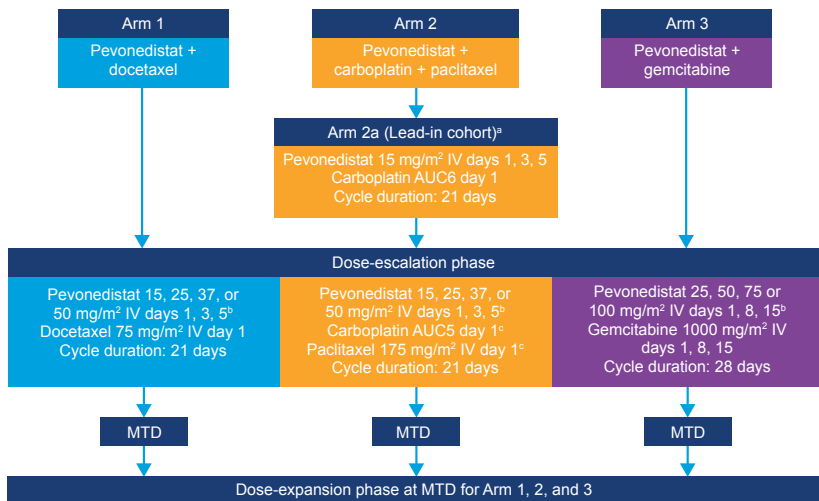

b

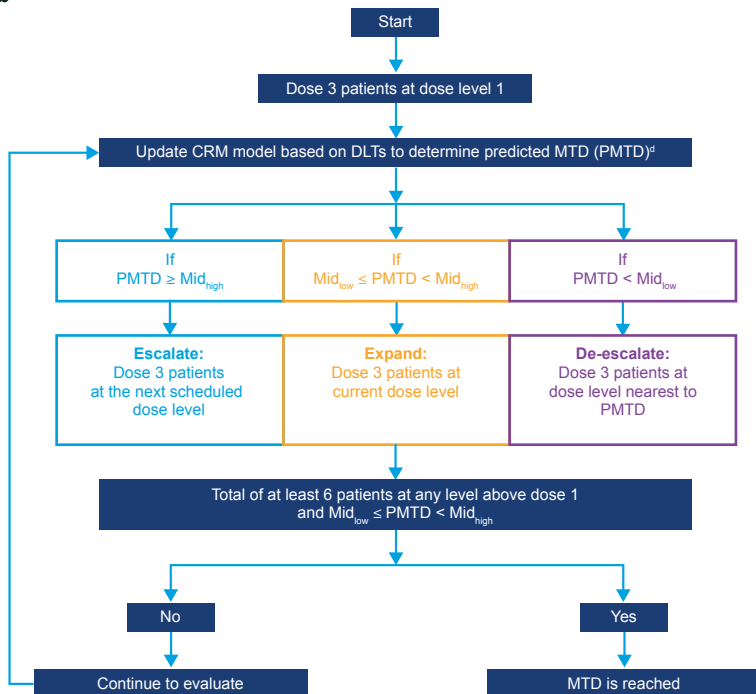

Supplement: Supplementary file 2 — (PDF 1335 kb) [file 10637_2018_610_MOESM2_ESM.pdf]
